# Impact of The Assist Ventilation Mode On Work of Breathing (Wob): Neurally Adjusted Ventilatory Assist (Nava) *Versus* Pressure Support Ventilation (Psv) *Versus* Proportional Assist Ventilation *Plus* (Pav+)

**DOI:** 10.1186/2197-425X-3-S1-A9

**Published:** 2015-10-01

**Authors:** R Di Mussi, S Spadaro, CA Volta, T Stripoli, A Armenise, L Pisani, RG Renna, A Civita, G Altamura, F Bruno, S Grasso

**Affiliations:** Università degli Studi di Bari 'Aldo Moro', Dipartimento dell'Emergenza e dei Trapianti d'Organo, Bari, Italy; Università di Ferrara, Department of Morphology, Surgery and Experimental Medicine, Ferrara, Italy

## Introduction

During assisted mechanical ventilation, appropriate WOB prevents diaphragm atrophy and fastens weaning. During PSV the ventilator support is fixed, irrespective of the patient's effort. On the contrary, both the NAVA and PAV + algorithms are designed to amplify patient's spontaneous effort. [1], [2]

## Objectives

To test if the assist ventilation mode algorithm has an impact on patient's WOB.

## Methods

In 12 patients NAVA, PSV and PAV+ were randomly applied in a crossover fashion, for 4 hours each. The electrical diaphragmatic activity (EAdi) was continuously recorded during all the modes. WOB per breath (WOB_BREATH_) was estimated by converting the EAdi pressure time product (PTP)/breath in muscular PTP/breath (PTP_BREATH_). The conversion factor was the ratio between EAdi peak and airway opening pressure negative peak during an end-expiratory occlusion.

## Results

See figure 1

**Figure 1:**
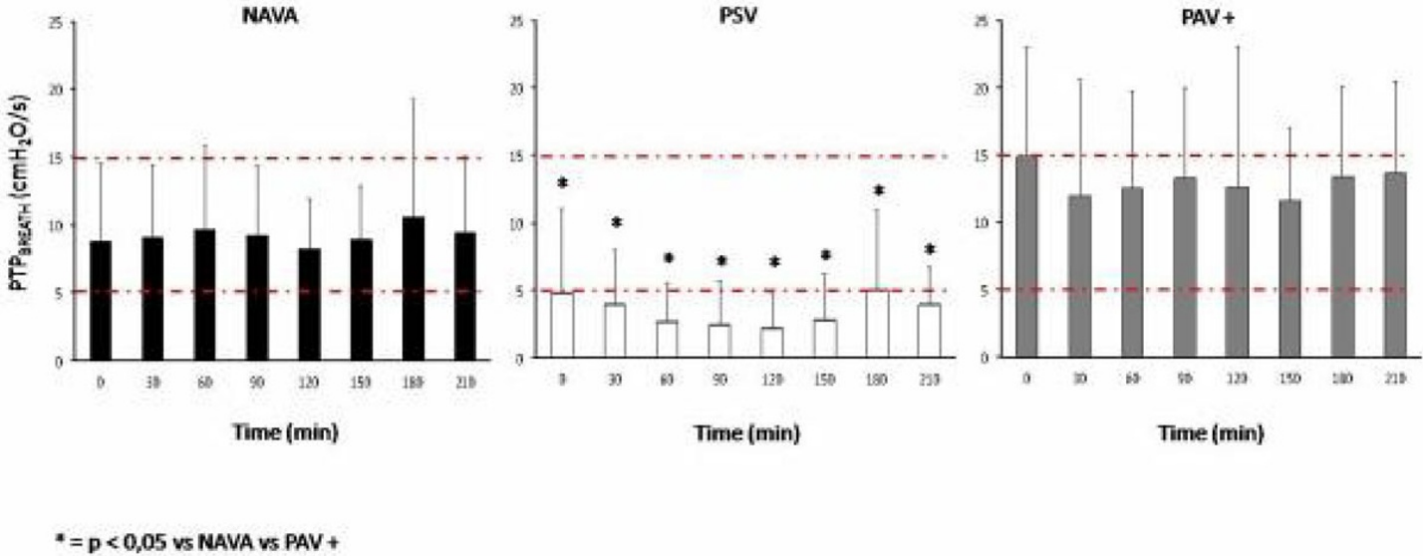
**PTP,breath vs time in the three ventilatory modes** **. Red lines: range of physiological PTP**_**BREATH**_

## Conclusions

PTP_BREATH_ was in the physiological range during NAVA and PAV + whereas during PSV remained constantly below it. We speculate that, as compared to PSV, NAVA and PAV+ favored a correct matching between diaphragm contraction and ventilator assistance. Further studies are required to test if the WOB pattern during assisted ventilation has an impact on mechanical ventilation duration and other clinically meaningful outcome parameters.
